# The influence of posture duration on hand tremor during tasks with attention-distraction in persons with Parkinson’s disease

**DOI:** 10.1186/s12984-019-0534-8

**Published:** 2019-05-28

**Authors:** Anne Sofie Bøgh Malling, Bo Mohr Morberg, Lene Wermuth, Ole Gredal, Per Bech, Bente Rona Jensen

**Affiliations:** 1 Department of Neurology, Odense University Hospital, University of Southern Denmark, Odense, Denmark; 2 Department of Clinical Research, University of Southern Denmark, Odense, Denmark; 3 The Danish Rehabilitation Centre for Neuromuscular Diseases, Taastrup, Denmark; 4 Psychiatric Research Unit, Psychiatric Centre North Zealand, University of Copenhagen, Hillerød, Denmark

**Keywords:** Parkinson’s disease, Rest tremor, Postural tremor, Accelerometry, Tremor intensity

## Abstract

**Background:**

Tremor is one of the hallmarks and most bothersome symptoms in Parkinson’s disease (PD). The classical PD tremor is present at rest, but postural tremor also occurs. PD tremor can be continuous or intermittently present and can have a re-emergent nature. The tremor intensity is affected by attention and stress level. Observations of PD tremor have indicated increased tremor intensity with time during 30-s tremor assessments. This phenomenon has not previously been studied systematically. Thus, in order to contribute to our understanding of the mechanisms associated with PD tremor, our aim was to investigate the influence of time during a posture holding and a resting task on hand tremor characteristics in persons with PD compared to healthy peers.

**Method:**

Fifty persons with PD and at least one tremoring hand (tremor intensity exceeding mean + 2SD of a healthy reference group (REF), *N* = 40) were included from a clinical trial population. Hand accelerations in a rest and postural condition were measured in 30-s assessments while the participants performed a self-paced simple subtraction task with eyes closed to standardize attention without inducing stress. Tremor intensity, maximal power, frequency of maximal power and tremor onset time was calculated for three consecutive 10-s time intervals.

**Results:**

Tremor intensity and maximal power increased significantly during the 30-s recording in the PD-group in both conditions (1st-3rd time-interval, tremor intensity: rest + 65% *p* < 0.0001, postural + 55% *p* < 0.0001; maximal power: rest + 93% *p* < 0.0001, postural + 82% *p* < 0.001). No effect of time was found on frequency of maximal power in the PD-group or on any effect measure in the REF-group.

**Conclusion:**

Tremor intensity and maximal power increased with time in the PD-group during 30-s tasks, while no change with time was found in the REF-group. In contrast, frequency of maximal power remained unchanged, which may suggest that the same neural circuits were responsible for the tremor generation throughout the tasks. The increase in tremor intensity and maximal power could not solely be explained by re-emergence of tremor. This suggests an increasing or gradually more synchronized cortico-spinal drive throughout the tasks. However, this requires further studies to determine.

## Introduction

Rest tremor is one of the hallmarks of Parkinson’s disease (PD) and is present in approximately 65% of the cases at disease onset and the prevalence increases with disease progression [1]. Persons with PD in the early disease stage (< 6 years from onset of symptoms) report tremor as the second most bothersome symptom [2]. Classical PD tremor has a dominant frequency of 4–7 Hz. Rest, postural and kinetic tremor of the upper extremities are common, while lower extremity tremor is rarer [1, 3].

PD tremor is a complex phenomenon and may be influenced by several internal and external factors. Re-emergent tremor, i.e. the short-delayed onset of tremor after repositioning of the limb, is a well-known phenomenon in PD [4]. In addition to the re-emergence phenomenon, tremor can be continuously present or have an intermittent nature where the intensity is highly variable across time with sudden increases and decreases in amplitude during both rest and postural conditions. Furthermore, an example of a PD postural tremor trace presented by Morrison et al. [5] indicated that tremor intensity increases with time over a 30s period. We observed a similar time-dependent increase in amplitude of both continuous and intermittent rest and postural tremor during recordings of pilot data (non-published). However, this phenomenon has to our knowledge not been studied systematically. Based on these findings, we hypothesized, that tremor intensity increases systematically with time during a posture holding or resting task.

PD tremor is influenced by attention and stress level [6]. Thus, tremor can in some cases deliberately be attenuated when attention is directed to it and stress amplifies PD tremor [6, 7]. Standardization of test conditions is therefore crucial when assessing PD tremor. Thus, several confounders and time-patterns of PD tremor has been reported emphasizing that tremor should be studied using a structured and systematic strategy to allow data interpretation and comparison between studies.

Our aim was to investigate the influence of time during a posture holding and a resting task on hand tremor characteristics in persons with PD compared to healthy peers and further to contribute to our understanding of the mechanisms associated with PD tremor as well as the significance of recording strategy and data analysis for the interpretation of outcome.

## Method

### Participants

The present study includes baseline accelerometer data sampled during a rest and a postural condition from a subsample of persons diagnosed with idiopathic PD (PD-group) participating in a double-blinded randomized clinical trial [8–11] (clinicaltrials.gov NCT02125032) along with data from an age and sex matched healthy reference group without visible tremor (REF-group) [8, 11, 12]. Inclusion criteria for the trial were an unchanged and optimal medical treatment regarding PD 6 weeks prior to participation; Mini Mental State Examination score > 22; and age > 18 years. Exclusion criteria were any known neuromuscular or neurological disease other than PD that might interfere with motor function; psychopathological treatment of other conditions than depression; substance abuse; and active medical implants. The REF-group was included to determine pathologic tremor threshold values defined as mean tremor intensity + 2 SD of the REF-group (rest: 0.300 m/s^2^, postural: 0.783 m/s^2^) [11] and to gain a reference pattern of time-dependency of movements in the two conditions. Fifty persons with PD had at least one tremoring hand and were included in this study. A tremoring hand was defined by having a rest or postural tremor intensity exceeding the tremor threshold. Only tremoring hands were included in the analyses. Group descriptive variables for the PD rest-tremor group, PD-postural tremor group and REF-group are listed in Table 1. The participants with PD followed their usual medication scheme on the day of assessment.

**Table 1:** Group descriptive variables

	N	N, females	N, hands	Age (years)	UPDRS motor	UPDRS total
PD, rest	36	17	52	66 (8.9)	26.5 (9.0)	46.2 (14.1)
PD, postural	43	19	64	65 (9.2)	25.2 (9.6)	45.8 (15.2)
REF	40	19	80	66 (1.3)	–	–

Mean (SD) of descriptive variables for the group of persons with Parkinson’s disease (PD) with rest and postural tremor, respectively, and the healthy reference group (REF). N reflects number of group individuals. N, hands reflects total number of included tremoring hands in the group. The Unified Parkinson’s Disease Rating Scale (UPDRS) motor and total score reflects motor symptom severity and disease severity respectively (higher scores representing higher severity).

### Protocol and accelerometer measurements

The procedure for the tremor assessment has been reported elsewhere [11]. In short, two-dimensional cylindrical accelerometers (Catsys PD, Danish Product Development Ltd., Snekkersten, Denmark) were fixed on the hand dorsum along and between metacarpal bone II and III on each hand and hand tremor was sampled synchronously from both hands at 50 Hz.

The participants sat on a chair with backrest and no arm support with their feet on the ground. 30-s assessments were performed twice in each of two conditions: 1) rest, while the hands were placed with palms down approximately on the middle of the thigh in a position allowing the participant to relax both hands and forearms and 2) postural, while the arms, hands, and fingers were extended in front of the body at shoulder height, approximate shoulder width between hands, and with palms facing towards the floor.

We attempted to standardize the attention level of the participants without inducing stress. Thus, the participants closed their eyes during the assessments to avoid distraction from the environment and performed a vocal serial subtraction task inspired by Lee et al. [7] to focus their attention on the subtraction task and not on the tremor assesment. The subtraction task was simplified to vocally count down from 100 in steps of two (instead of 7 or 8) in a self-paced manner (instead of as quickly as possible) to avoid stress. Before each assessment, it was emphasized that the participant should sit as calm and relaxed as possible. The assessments were performed in calm surroundings and in the order rest, postural, rest, postural with approximately 30-s pauses between assessments.

### Data analysis

Data analyses were performed in MATLAB (Mathworks Inc., USA). The resultant acceleration was calculated and the 30-s time series were divided into three equal time intervals of 10 s. For each 10-s time interval, the following measures were calculated within the frequency band of 3–8 Hz (0.1 Hz frequency resolution):

- *Tremor intensity* was calculated as $\sqrt{\frac{\sum_{3\  Hz}^{8\  Hz}{\left| fft\right|}^2}{N^2}}$, where |*fft*| is the absolute value of the fast Fourier transformation of the resultant acceleration in the 3–8 Hz band, and N is the number of |*fft*| observations. According to Parseval’s theorem, this corresponds to the root mean square of the resultant acceleration within the 3–8 Hz frequency band.
- *Maximal power* was the maximal absolute power within the 3–8 Hz band.
- *Frequency of maximal power* was the frequency at which the power was maximal.

To investigate the time of tremor onset, we made a running calculation of the tremor intensity for each 2-s period moving 0.02 s for each calculation. Tremor onset was defined as the first 2-s period with a tremor intensity above the previously defined threshold. Furthermore, we calculated the slope of the least-square best-fit linear regression of the tremor intensity as a function of time from tremor onset and to the end of the 30-s acquisition period.

### Statistics

Statistics were performed in SAS 9.4 using linear mixed models (the log transform of tremor intensity and maximal power were used for analyses to meet normal distribution). The level of significance was set to 0.05.

We investigated if the intensity related measures were dependent on time interval (0–10s, 10–20s, 20–30 s) and if a dependency differed between groups (PD vs REF). The model used was:

Effect measure = group _^_ time-interval _^_ group × time-interval with a compound symmetry covariance structure differentiated between groups and with *time-interval* categorizing repeatedly measured effect measures and considering participants as random factor to meet the non-independence between hands of the same participant. In case of significant *group* × *time-interval* interaction, pairwise comparison within groups across intervals and between groups within intervals were performed and a 3-level Bonferroni correction was applied to adjust for multiple comparisons (corrected significance level of 0.017).

A sign test was assigned to the slope of the least-square best-fit linear regression of tremor intensity.

Means and standard deviations (SD) of normally distributed variables are presented, while medians and the inter quartile ranges (IQR; indicating the middle 50% of the data) of non-normal distributed variables are presented.

## Results

**Fig. 1:**
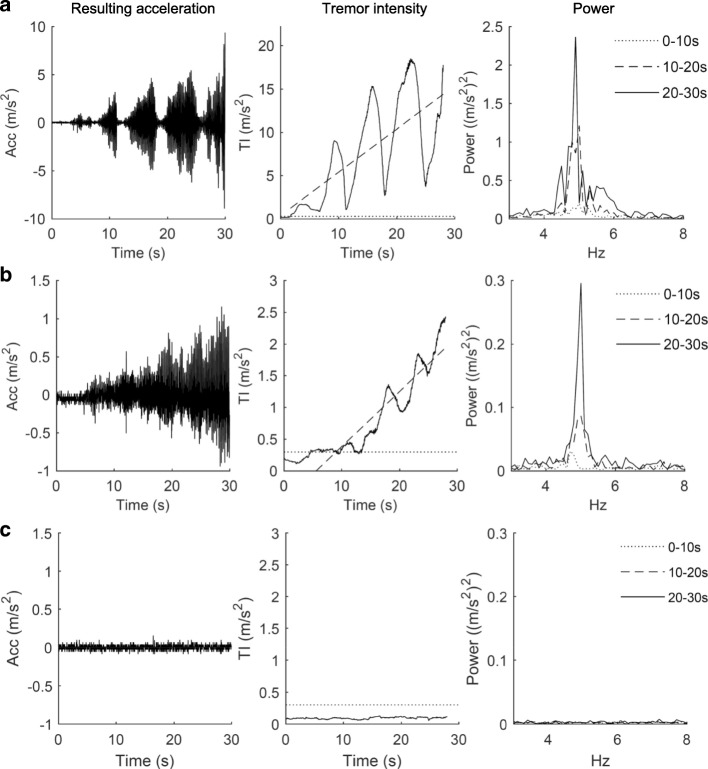
Examples of rest tremor. The time course of resulting acceleration (Acc) (left column), tremor intensity (TI) (middle column) and the power spectrum for each 10s interval (right column) for a participant with Parkinson’s disease having substantial intermittent tremor (row **a**), a participant with Parkinson’s disease having mild continuous tremor (row **b**), and a reference participant (row **c**). The signal-to-noise ratio is low in row **c** (left column) due to very low intensity or absence of hand movements in the reference participant. Note that axis scales in row **a** are different from row **b** and **c**. In the TI plots, the intensity-limit of rest tremor (mean TI of REF + 2 SD) is indicated by a dotted line and the least-square best-fit linear regression of TI(t) is indicated by a truncated line

Different patient-specific time-pattern of tremor intensity was seen during the 30-s recording. Some participants showed continuous tremor pattern while other showed a much more variable or intermittent pattern across time (Fig. 1).

By using objective criteria for initiation of tremor, i.e. exceeding mean tremor intensity of REF + 2SD, we found that the mean latency for tremor onset was 1.8 s and 1.4 s in the rest and postural condition respectively. All PD hands, except two, had tremor onset within the first time interval (0–10 s) in both the rest and postural condition. In the rest condition, 65% had tremor present at the start of the 30 s measuring period, while this was the case for 55% in the postural condition.

**Fig. 2:**
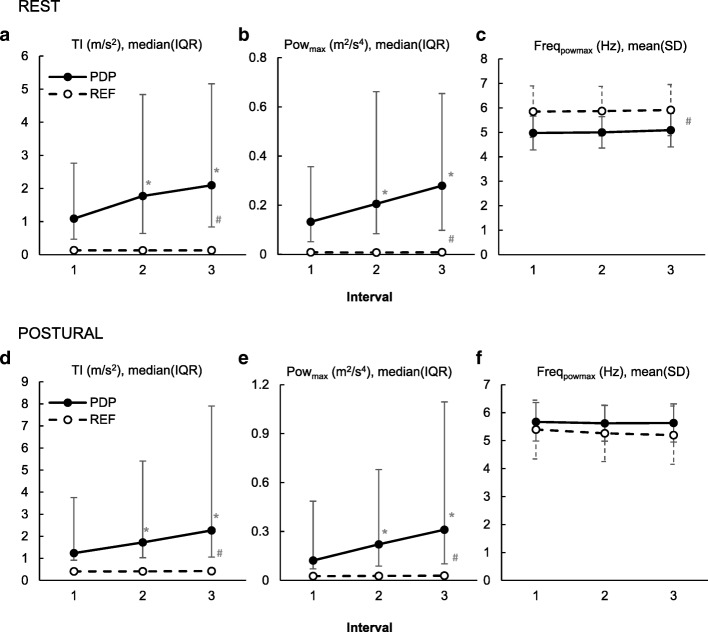
Group effect of time intervals for the rest and postural condition. **a**&**d** Tremor intensity (TI). **b**&**e** Maximal power (Pow_max_). **c**&**f** Frequency of maximal power (Freq_powmax_). PD = Parkinson’s disease group. REF = healthy reference group. Due to the homogeneity of the REF-group, the inter quartile range (IQR) of the TI and Pow_max_ are very low and thus concealed by the markers. * = significant within group difference from interval 1 (*P* ≤ 0.0167). ^#^ = significant difference between groups at all intervals (P ≤ 0.0167).

We found significant *group* × *time-interval* interactions in both conditions on tremor intensity (rest *p* < 0.001, postural *p* < 0.001) and maximal power (rest *p* < 0.001, postural *p* < 0.001). Tremor intensity as well as maximal power increased significantly during the 30-s recording in the PD-group (model estimate of median increase from 1st to 3rd interval, tremor intensity: rest + 65% *p* < 0.0001, postural + 55% *p* < 0.0001; maximal power: rest + 93% *p* < 0.0001, postural + 82% *p* < 0.0001) while the frequency of maximal power remained unchanged (*group* × *time-interval*: rest *p* = 0.9368, postural *p* = 0.6763). Accordingly, we found that the tremor intensity increased after tremor onset (rest: median (IQR) of tremor intensity slope = 0.0188 (0.0058–0.0849) m/s^3^, *p* < 0.001; postural: median (IQR) of tremor intensity slope = 0.0173 (0.0007–0.1325) m/s^3^, *p* < 0.001). In contrast, no changes in tremor intensity, maximal power, or frequency of maximal power with time were found for the REF-group (Fig. 2).

## Discussion

The tremor intensity increased systematically during the 30-s measurement period in the PD-group, whereas the tremor intensity remained constant throughout the duration of the tasks in the REF-group. In accordance, an increase in maximal power with posture duration in the PD-group at both the resting and the postural condition was found. However, the frequency of maximal power remained the same across the time intervals. This may suggest that the same neural circuits were responsible for the generation of tremor throughout the acquisition despite the increased intensity.

The increased tremor intensity and maximal power with prolonged posture duration in the PD group may be explained by 1) a re-emergent nature of the tremor with a delayed onset of tremor after the start of the 30-s assessment and/or 2) a continuous amplification of tremor amplitude during the task. Tremor onset time was determined to explore if the increased tremor intensity with time could be explained by postponed onset of tremor. The results showed that tremor was initiated within the first 10-s interval in all but two hands and with a mean onset time of 1.8 s and 1.4 s in the rest and postural condition, respectively. Previous studies have found a longer (9–10 s) mean latency of re-emergent tremor [4, 13]. This difference in latency may be attributed to the counting task applied in the present study. Drawing attention to the tremor can attenuate the intensity or even stop the tremor. Thus, as the attention was directed towards the counting task, the participants were probably less prone to consciously or unconsciously suppress the tremor. Another plausible explanation for the difference in latency is the criterion for tremor onset. In the present study, we used an objective criterion based on a healthy reference group, whereas previous studies have used visible detection of tremor as the onset criterion [4, 13]. Our data showed that the increase in tremor intensity from time-interval 1 to 2 was to some extend influenced by the successive onset of tremor, and that this occurred primarily in the beginning of the first time-interval.

The remaining increase in tremor intensity must be attributed to an amplification of tremor amplitude, which was supported by our finding of a significantly positive slope of the best fit linear regression of tremor intensity after tremor onset. Thus, our results showed that both tremor onset and amplitude amplification occur during prolonged posture duration. A dual origin of tremor is in accordance with the dimmer-switch model of PD tremor proposed by Helmich and colleagues. Based on electromyographic and functional magnetic resonance imaging studies, they found that the basal ganglia were transiently activated at the onset of tremor episodes, whereas activity of the cerebello-thalamo-cortical circuit was associated with the magnitude of tremor in terms of electromyographic amplitude [14]. Thus, the model proposes a “switch” effect of the basal ganglia (more specific the internal globus pallidus) initiating hand tremor whereas a cerebello-thalamo-cortical circuit functions as a “dimmer” regulating tremor amplitude [14, 15]. Based on the findings that parkinsonian rest tremor amplitude remained unchanged despite of spontaneous changes in tremor frequencies caused by deep brain stimulation of the ventrolateral nucleus at near-to tremor frequencies [16], Helmich and colleagues have suggested, that within the cerebello-thalamo-cortical circuit, the motor cortex has a specific role in determining tremor amplitude whereas the thalamus and cerebellum may be important for maintenance of the tremor frequency [17]. Therefore, we hypothesize that the systematic increase in tremor intensity with time reflects an increased or more synchronized muscle activation elicited by altered cortico-spinal drive to the upper extremities. However, the exact mechanism responsible for the continuous amplification needs to be explored in future studies and could be seen as an extension of the dimmer-switch model.

Recently, Dirkx et al. proposed that postural tremor in PD consist of two distinct phenotypes; 1) the most common (≈80%) re-emergent tremor characterized by amplitude depression right after repositioning of the limb, a small frequency difference from rest tremor, and a clear dopamine dependency, and 2) the rarer (≈20%) pure postural tremor characterized by no amplitude suppression with repositioning of the limb, a larger frequency difference from rest tremor (+ 3.5 Hz), and no dopamine dependency [18]. The present study focused on the 3–8 Hz frequency range and is therefore expected primarily to represent the most common re-emergent postural tremor according to the classification by Dirkx et al.

It may be noted, that recruitment of additional motor units and increased synchronization between motor units is expected during muscle fatigue leading to decreased steadiness and higher tremor intensity during isometric contractions [19, 20]. This phenomenon is, however, not likely to have influenced our findings of increased tremor intensity with time, since the duration of the posture holding was short (30 s) and performed with no external load, and since the time-related increase of intensity was also found in the resting condition. Electromyographic assessments were not performed in the present study, although they might have contributed further to our understanding of the neural mechanisms of aggregated tremor.

Proper quantification of tremor is of clinical importance to ensure reliable evaluation of treatment interventions and to initiate the most appropriate treatment [21]. Our results contribute to understanding of the complexity of tremor assessment by showing that tremor is also highly time dependent within a short-duration task, emphasizing the importance of strictly standardized test conditions. From an analytical point of view, it is important to quantify the re-emergence, the intermittency (in some individual) and the amplification of tremor after re-emergence objectively. This can be done by standardizing the onset of acquisition in relation to repositioning of the hands or limbs, and by acquiring enough data to describe the cyclic intermittent tremor bursts and the time-dependent amplification of tremor intensity. Thus, we suggest acquiring accelerometer data for at least 30 s and preferably longer.

## Conclusion

The main findings of the present study were that tremor intensity and maximal power increased with time in the PD-group during 30-s tasks, while no change with time was found in the REF-group. In contrast, the frequency of maximal power remained unchanged, which may suggest that the same neural circuits were responsible for the tremor generation throughout the tasks.

The increase in tremor intensity and maximal power could not solely be explained by re-emergence of tremor. This suggests an increasing or gradually more synchronized cortico-spinal drive to the arm muscles throughout the tasks. However, this requires further studies to determine.

## Abbreviations

IQR: Inter-quartile range
PD: Parkinson’s disease
REF: Healthy reference participants
